# ID 294 - Regulação do Acesso a Consultas e Serviços de Apoio Diagnóstico e Terapêutico em Sistemas Públicos de Saúde: uma revisão de escopo assistida por inteligência artificial

**DOI:** 10.5327/2237-9622.2025.v34s1.294

**Published:** 2025-11-25

**Authors:** Tiago Silva Vaz, Leila Bernarda Gottems

**Keywords:** regulação em saúde, Atenção Secundária à Saúde, assistência ambulatorial

## Abstract

**Introdução:**

A regulação do acesso a consultas e serviços de apoio diagnóstico e terapêutico (SADT) é um dos principais desafios enfrentados por sistemas públicos de saúde em todo o mundo. A crescente demanda por serviços especializados, aliada à escassez de recursos e à desigualdade no acesso, gera dificuldades no gerenciamento eficiente dos fluxos assistenciais. Este estudo tem como objetivo realizar uma revisão de escopo com a seguinte pergunta: quais conhecimentos e evidências estão disponíveis na literatura quanto à regulação do acesso a consultas e serviços de apoio diagnóstico e terapêutico em sistemas de saúde?

**Método:**

Esta revisão de escopo foi conduzida seguindo as diretrizes do PRISMA-ScR (). A busca foi realizada em bases de dados científicas como PubMed, Scopus e SciELO, utilizando termos relacionados à "Atenção Secundária à Saúde", "Assistência Ambulatorial", "Listas de Espera", "Agendamento de Consultas" e "Técnicas e Procedimentos Diagnósticos". Os artigos não foram limitados pela lingua, pelo país ou pelo ano de publicação. O ChatGPT foi usado como subsídio para construir conceitos e correlação dos artigos com a literatura.

**Resultados:**

Dos 1.668 artigos identificados inicialmente, 105 foram incluídos nesta revisão. Os estudos analisados buscaram investigar quais tipos de intervenção foram adotados para melhorar os encaminhamentos e o acesso oportuno. Ao final, os artigos foram conceituados e divididos em três categorias: intervenções para melhorar o encaminhamento da Atenção Primária à Secundária (Apae); na Atenção Ambulatorial Especializada (AAE) e no Percurso Assistencial do Paciente (Pasp); sendo o maior número de artigos relacionados à categoria Apae.

**Conclusão:**

A revisão demonstra que existem inúmeras intervenções propostas com o objetivo de aumentar o acesso a consultas e SADT em sistemas públicos que perpassam por descrição e ajustes no serviço, tempo de espera, avaliação de barreiras e desafios, sistemas de coordenação e colaboração entre os níveis de atenção, abordagens holísticas e aplicação de protocolos e diretrizes assistenciais.

A regulação do acesso a serviços de saúde é uma questão complexa que exige soluções inovadoras. A revisão aponta que intervenções específicas, quando bem planejadas e implementadas, podem melhorar não apenas a eficiência e a qualidade, mas também a equidade do atendimento, especialmente em contextos de saúde pública em que esses fatores são críticos. Futuras pesquisas devem explorar mais profundamente os impactos, a viabilidade e a aplicabilidade das intervenções em diferentes contextos de sistemas públicos de saúde.

